# Making sense of phantom limb pain

**DOI:** 10.1136/jnnp-2021-328428

**Published:** 2022-05-24

**Authors:** Hunter R Schone, Chris I Baker, Joel Katz, Lone Nikolajsen, Katleho Limakatso, Herta Flor, Tamar R Makin

**Affiliations:** 1 NIMH, National Institutes of Health, Bethesda, Maryland, USA; 2 Institute of Cognitive Neuroscience, University College London, London, UK; 3 Department of Psychology, York University, Toronto, Ontario, Canada; 4 Transitional Pain Service, Department of Anesthesia and Pain Management, Toronto General Hospital, Toronto, Ontario, Canada; 5 Department of Clinical Medicine, Aarhus University, Aarhus, Denmark; 6 Department of Anesthesiology and Intensive Care, Aarhus University Hospital, Aarhus, Denmark; 7 Department of Anaesthesia and Perioperative Medicine, Pain Management Unit, Neuroscience Institute, University of Cape Town, Rondebosch, Western Cape, South Africa; 8 Institute of Cognitive and Clinical Neuroscience, Central Institute of Mental Health/Medical Faculty Mannheim, Heidelberg University, Mannheim, Germany; 9 Center for Neuroplasticity and Pain, Department of Health Science and Technology, Faculty of Medicine, Aalborg University, Aalborg, Denmark

**Keywords:** PAIN, BRAIN MAPPING, COGNITION, SOMATISATION DISORDER, MOTOR CONTROL

## Abstract

Phantom limb pain (PLP) impacts the majority of individuals who undergo limb amputation. The PLP experience is highly heterogenous in its quality, intensity, frequency and severity. This heterogeneity, combined with the low prevalence of amputation in the general population, has made it difficult to accumulate reliable data on PLP. Consequently, we lack consensus on PLP mechanisms, as well as effective treatment options. However, the wealth of new PLP research, over the past decade, provides a unique opportunity to re-evaluate some of the core assumptions underlying what we know about PLP and the rationale behind PLP treatments. The goal of this review is to help generate consensus in the field on how best to research PLP, from phenomenology to treatment. We highlight conceptual and methodological challenges in studying PLP, which have hindered progress on the topic and spawned disagreement in the field, and offer potential solutions to overcome these challenges. Our hope is that a constructive evaluation of the foundational knowledge underlying PLP research practices will enable more informed decisions when testing the efficacy of existing interventions and will guide the development of the next generation of PLP treatments.

## Introduction

Now more than ever, scientists and clinicians are finding it challenging to determine the reliability of published evidence. In research, this issue is exacerbated by an increase in publications producing an overwhelming barrage of statements, not all of which are equally supported by sufficient evidence. As such, it is becoming increasingly more challenging to distinguish well-established facts from substandard evidence and speculations. Within this research climate, there are multiple reasons why, despite a growing body of research and review on phantom limb pain (PLP), there has been little convergence in understanding the nature of this peculiar condition. Individuals who undergo limb amputation commonly report feeling painful sensations perceived to originate from the missing limb.^1^ Despite multiple theories on what causes PLP, there is still no clear consensus on the underlying mechanisms driving PLP^2^ or treatment approaches.^3 4^ As such, the dozens of pharmacological and non-pharmacological treatments^4 5^ operate on a variety of targets across the neural axis^6^ with limited success. So, why, after nearly 75 years of research, is there still so little consensus on PLP?

While there have been several efforts to comprehensively review PLP literature,^2 4 7 8^ there remains poor consensus on what research practices are necessary to accumulate reliable knowledge on PLP. As such, we believe there is a need to first reach an agreement on the main research challenges hindering progress and then to provide potential solutions for building a better foundational understanding of the clinical characteristics of PLP and their underlying mechanisms. Beyond the need to encourage a more critical assessment of PLP research methodology, we are writing this review to empower researchers and clinicians with the tools necessary to more reliably evaluate and execute this research. Toward this goal, we first discuss challenges of pain research and PLP in particular, which impact our ability to reliably assess prevalence and heterogeneity of phantom phenomena in amputee populations. Next, we consider the demographic and amputation-related factors associated with PLP and how these factors of interest help contribute to our understanding of PLP mechanisms. We next turn to clinical efficacy of behavioural PLP treatments and address the gap between primary research evidence and clinical practice. Finally, we suggest how common research practices could be improved to generate a better conceptual and technical basis for PLP treatment. While the focus of our review is on improving PLP research, the issues and potential solutions we highlight are likely to be relevant to a range of related difficult to treat pain conditions (eg, complex regional pain syndrome, fibromyalgia and lower back pain).^9^

### Challenges in studying PLP

A major challenge with studying PLP is that, as a pain condition, it is a multifaceted experience which reflects the combined sensory, emotional and cognitive domains. How pain is experienced is modulated by multiple dynamic factors unique to each individual, such as genetics,^10^ psychological factors (stress, anxiety and attention, to name a few),^11^ pain-related cognitions (eg, perceived control over pain),^12^ social-environmental factors (eg, social support)^12^ and even cultural circumstances.^13^ Beyond the general issue of quantifying a pain experience, PLP introduces several additional intrinsic challenges. First, unique to PLP, the pain is perceived to originate from a body part that is no longer physically present, which complicates the sensory, emotional and cognitive associations with the experience, influencing the nature of how the pain is reported (even relative to other pain conditions). Second, PLP has a complex phenomenology with multiple covariates and rarely occurs without other amputation-related sensations (eg, residual limb pain (RLP), non-painful phantom sensations) which may or may not be relevant to PLP prevalence. Finally, PLP is not only heterogenous in the nature of the experience (quality, intensity, frequency, time of onset, etc) but also in the various reasons for amputation (traumatic, infection, cancer, vascular disease),^14^ stage of life (eg, in paediatric amputees and throughout adulthood)^15 16^ and body part amputated (extremity and level of amputation).^17^ Therefore, any proposed theory for what causes PLP and how to treat it will need to consider its multifaceted phenomenology and heterogeneity.

A second challenge to PLP research relates to common methodological problems. While these issues, too, are not unique to PLP, their impact on the strength of evidence for PLP research studies is fundamental, starting with how PLP is quantified. In particular, PLP treatment outcomes are measured by self-report, which is known to vary over time and context.^18^ Currently, individual studies use a variety of measures to quantify PLP, such as different scales (eg, Numerical Rating Scale; Universal Pain Score; Visual Analogue Scale (VAS); FACES Rating Scale; Patient Reported Outcomes Measurement Information System, for a review on pain scales),^19^ pain diaries^20^ and questionnaires (eg, McGill Pain Questionnaire (MPQ); 36-Item Short Form Survey (SF-36); West Haven-Yale Multidimensional Pain Inventory; Groningen Questionnaire Problems after Leg/Arm Amputation; Chronic Pain Grade). Additionally, instructions to participants to assess their pain over various time windows introduces heterogeneity in prevalence estimates because PLP researchers typically are not well-versed in epidemiological methods and so do not distinguish between different prevalence estimates (eg, point prevalence: ‘Do you have PLP right now?’, period prevalence: ‘Have you had PLP in the last X months?’ or lifetime prevalence: ‘Have you ever had PLP?’). PLP ratings are collected via different platforms (self-administered questionnaires; phone surveys; pain diaries; interviews with experienced clinicians). Further, each of these measures is designed to probe slightly different features of the pain experience. This diversity of methods hinders the collation of evidence across studies. But even more problematic, without careful a priori selection of the most relevant outcome measures for a given study, each study will collect a range of different PLP-related measures, creating opportunities for ‘p hacking’,^21^ when a range of different PLP related measures are administered and the outcome with the best statistical results is reported. Finally, adding the relatively low prevalence of amputation in the general population and high heterogeneity in the PLP experience and amputation-related factors results in too many variables and too few participants for adequate statistical power. Taken together, these methodological and statistical issues make it likely that spurious findings from individual studies may bias and even offset, our understanding of the nature of PLP and, subsequently, its treatment. In the face of these challenges, a crucial first step to developing a better understanding of PLP is characterising the prevalence and heterogeneity of the phenomena, as well as associated amputation-related factors. This fundamental background can help hint at the underlying mechanism(s) of PLP and, consequently, help to scrutinise the rationale for modern PLP treatment approaches. In the next sections, we will address each of these in turn.

### Characterising the prevalence and heterogeneity of PLP

**Figure 1:**
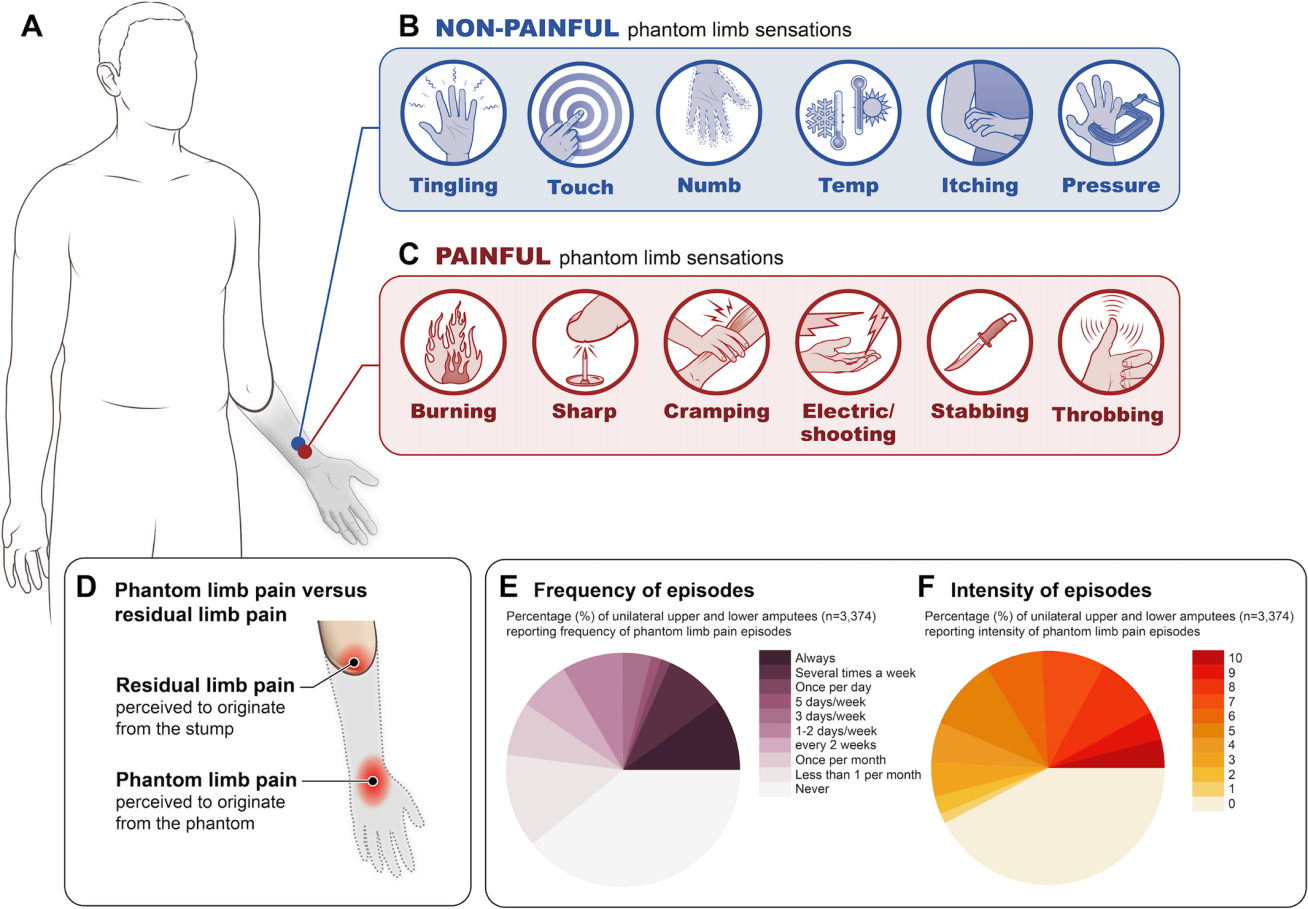
Phantom limb sensations. (A) Diagram of upper limb amputee with a phantom arm. Spectrum of phantom sensations from non-painful (B) to painful (C). (D) Residual limb pain (RLP) and PLP illustrated in an upper limb amputee. RLP is perceived to originate from the stump/residual limb, whereas PLP is pain perceived to originate from the phantom limb. (E) Cross-sectional surveys on PLP frequency (data adapted from figure 2 in Diers *et al* 32 with permission) and (F) average intensity (data adapted from figure 2 in Diers *et al* 32 with permission). Illustrations and remade figures are original.

PLP is an umbrella term for a hugely heterogenous phenomenon. After limb amputation, most individuals experience a phantom limb and perceive painful sensations to originate from it (figure 1A, B). PLP sensations are often reported as sharp, electric-shock-like, shooting, stabbing, throbbing, burning, aching and/or cramping pains (figure 1C).^1 22–25^ For example, one amputee who had an arm and leg amputated as a result of a motorcycle accident described their phantom pain ‘as if the skin of my arm has been ripped off; salt is being poured on it and then it’s thrust into fire. I also sometimes feel as if the fingers on my amputated hand are moving uncontrollably’.^26^ Another described their pain as ‘difficult to explain. It’s as if I am lying in a nest of insects, and they’re constantly crawling not only outside but inside my body’. More than the quality of the pain, there is a strong consensus that most amputees experience some form of PLP, though there is very little knowledge on the incidence of PLP (number of new cases per unit time) due to the paucity of longitudinal studies.

Across several recent large-sample surveys,^27–32^ systematic reviews^15^ and meta-analyses,^33^ estimates of PLP prevalence are highly varied, in large part, because researchers are not specific about the time frame over which the participants are asked. Moreover, how PLP prevalence is defined will produce different rates even when using data from the same sample. As such, reports of PLP prevalence should be interpreted with caution. In particular, a recent meta-analysis of 39 studies, comprising 12 738 upper-limb and lower-limb amputees using a mixture of prevalence measures, estimated an overall PLP prevalence rate of 64% (95% CI: 60.01 to 68.05), unsurprisingly with high statistical heterogeneity across studies.^33^ Pooling prevalence estimates can lead to high statistical heterogeneity across studies, not necessarily due to sampling error, but due to the different prevalence time windows assessed.^15^ Simply put, there may be no such thing as a ‘typical’ PLP phenomenon. For example, the frequency and intensity of PLP varies between individuals and within individuals over time (figure 1E).^32^ Further, pain intensity shows considerable variability with amputees reporting pain intensity to range from none (42%), mild (VAS 1–3: 9%), moderate (VAS 4–7: 32%) to severe (VAS 8–10: 17%; figure 1F).^32^

There are also a few reports that cite extremely low PLP prevalence estimates.^34–36^ For example, in a survey of lower limb amputees in Bahrain, only 1 of 45 amputees reported PLP.^34^ In a study of 391 amputees in China, only 29% of the amputees reported experiencing PLP.^36^ These outlier estimates may potentially be influenced by genetics or cultural circumstances, where people may be reluctant to report their pain, due to the fear of being stigmatised as mentally ill.^37^ These and other social and practical considerations may contribute to variability in global PLP prevalence and incidence estimates.

While differences in the quality of PLP is key to shaping the patient’s experience and quality of life, it is very common for researchers to ignore these dimensions of pain and instead use a dichotomous measure (PLP present or absent). Therefore, more comprehensive criteria for assessing PLP that incorporate the heterogeneity in pain onset, frequency, quality, duration and intensity, as well as its impact on quality of life (eg, sleep and daily functioning), are essential for future research.

### Factors associated with PLP and links to potential mechanisms

Given the covariation of PLP with other factors, including amputation-related factors, it is crucial to identify which factors are reliably associated with PLP and elucidate the mechanism(s) that link these factors to PLP. This requires prospective longitudinal designs with large samples. Moreover, identification of a risk factor^38^ requires a prospective study and one in which the putative risk factor is measured before the onset of PLP, that is, before amputation. But, prospective studies, starting prior to amputation, are scarce,^39–41^ with most studies using a cross-sectional design or retrospective design. This means the quality of available evidence for PLP association factors, including those detailed below, falls below this epidemiological gold standard.

**Figure 2:**
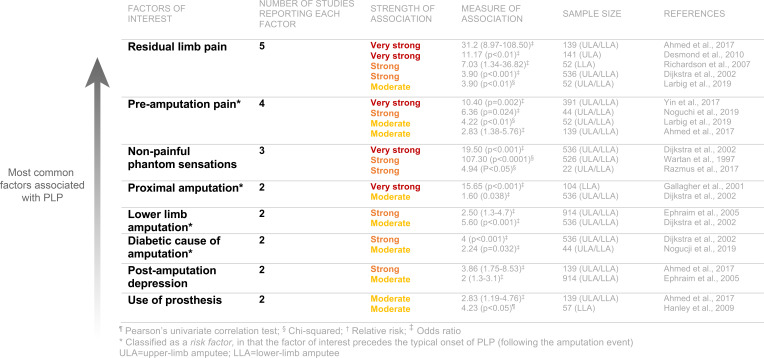
Factors positively associated with phantom limb pain (PLP). Most common factors associated with PLP identified in the meta-analysis by Limakatso *et al*.^33^ In selecting these factors, we chose to report those that met the following two criteria: reported in at least two studies and have a positive association that was moderate to very strong in strength. Data adapted from Table 3 in Limakatso *et al*., 2020 with permission.

To mitigate some of the methodological issues discussed so far, meta-analyses and systematic reviews incorporate samples across multiple studies. Combining samples provides a potential means to control for high sample heterogeneity across studies, but it is critical to remember that such analyses do not avoid the inherent limitations and biases introduced in the original studies. Indeed, if the evidence from the original studies is unreliable, the conclusions from the meta-analyses will also be unreliable (a problem termed ‘garbage in, garbage out’).^42^ In a recent meta-analysis, Limakatso and colleagues^33^ reviewed the evidence from 15 studies (mostly cross-sectional) with a total of 4102 amputees and identified 25 candidate factors potentially associated with PLP (figure 2). While this meta-analysis simply identified that many potential associations exist, the strength of the evidence supporting each of these factors varies widely. The three factors with the strongest level of evidence supporting an association with PLP across the greatest number of individual studies were RLP, preamputation pain and non-painful phantom sensations. Next, we discuss each of these factors in turn, as well as time since amputation which is commonly thought to be associated with PLP. For each of the factors of interest, we discuss how they help contribute to our understanding of PLP mechanisms, the primary purpose being to start to construct a more comprehensive characterisation of PLP.

#### Residual limb pain

**Figure 3:**
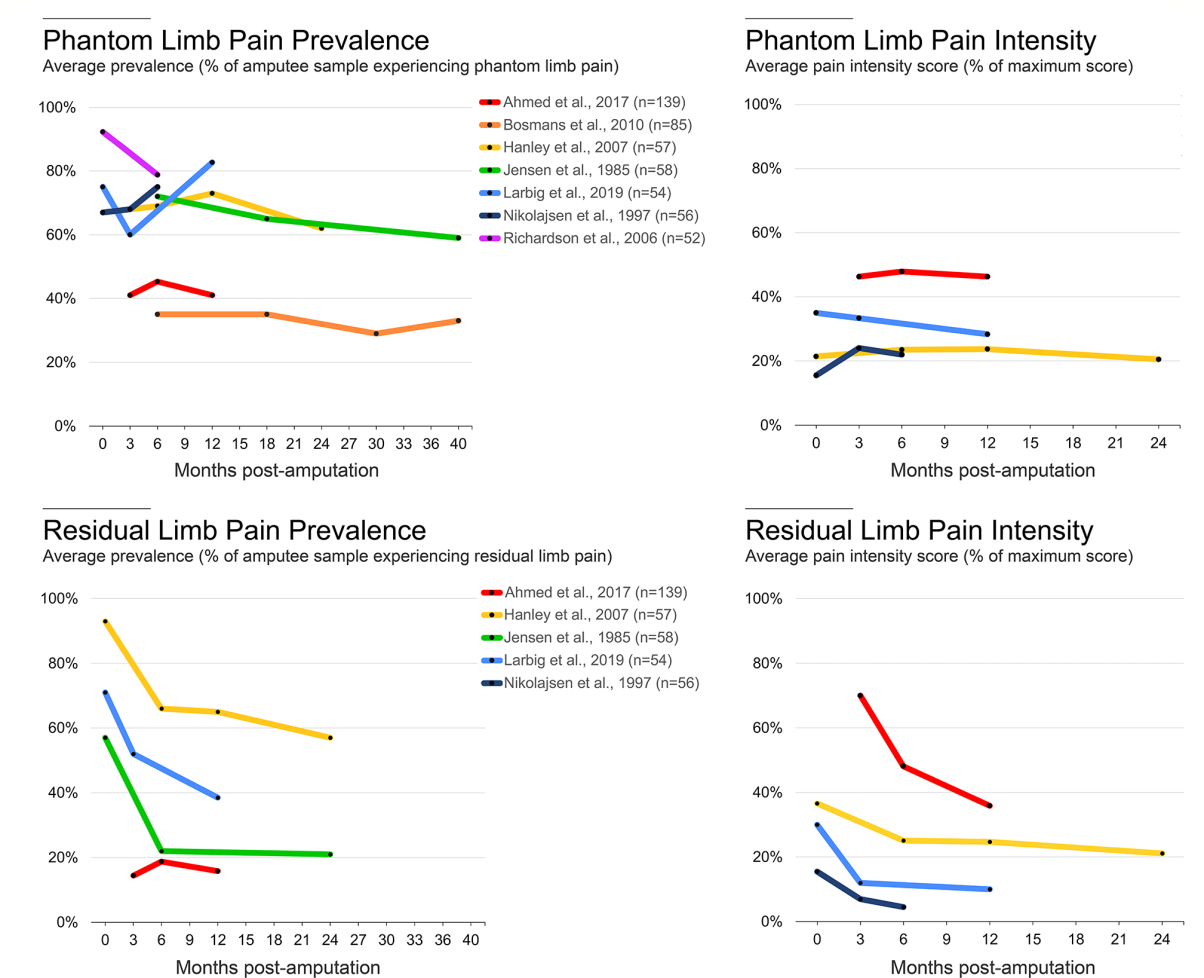
Time course of phantom limb pain (PLP) and residual limb pain (RLP). To characterise the time course of postamputation pain, we identified all longitudinal, peer-reviewed studies that report prevalence rates and average pain intensity values for PLP and RLP at multiple time points. In the top row, based on PLP data from prospective longitudinal studies, the number of individuals who report PLP and the intensity scores remains relatively constant with time. In the bottom row, based on RLP data from the longitudinal surveys, there is a large decrease in the number of individuals reporting RLP and in the intensity of RLP experienced in the first 6 months after amputation. Black dots represent individual timepoints. Colours depict individual studies; n values report the amputee sample sizes of each study. Patient drop-off across timepoints is not reflected in the starting sample sizes listed after references. The data used to generate these plots and a description for how the data were acquired are available at OSF (https://osf.io/3u8b4/).

The most common factor positively associated with PLP is RLP (identified in five studies, representing a total of 920 participants). RLP is described as pain perceived to originate from the remaining portion of the limb after amputation, most often close to the site of amputation (figure 1D). Amputees typically describe RLP as distinct from PLP, though RLP should not be viewed as a minor secondary pain condition relative to PLP. For example, in one survey, 33% of amputees reported RLP as the most problematic pain, followed by PLP (24%).^23^ Additionally, prevalence estimates of RLP have been shown to be similar to PLP.^23 27 30^ Considering the high covariation between PLP and RLP, it is essential to understand whether these two pain phenomena relate to each other mechanistically. An interesting potential dissociation between these related forms of pain is that over the first months following amputation, RLP tends to decrease—most likely due to the resolution of postoperative surgical wound pain—while chronic PLP remains consistent (to be discussed; figure 3).

This close association between RLP and PLP likely points to the potential contribution of peripheral factors in the onset of PLP such as the state of the residual nerve endings after ligation and transection,^43^ molecular alterations throughout the nerve triggered by the nerve transection^44^ or ectopic nerve impulses originating from a neuroma or the dorsal root ganglion (DRG).^45 46^ Regardless of the specific peripheral site (eg, neuroma, injured axons, DRG), the mechanistic view of these peripheral origins of PLP is that treatment should aim to silence these aberrant inputs so that they do not reach the central nervous system. Recent innovations in nerve transection procedures (eg, targeted muscle reinnervation^47^; agonist–antagonist myoneural interface^48^; regenerative peripheral nerve interfaces)^49^ have been proposed to reduce PLP by addressing one or more of the peripheral factors noted above. However, the current evidence is not sufficient to support a clear role for these novel peripheral nerve procedures in PLP alleviation.

#### Preamputation pain

According to the meta-analysis by Limakatso and colleagues,^33^ preamputation pain was the second most common factor positively associated with PLP (identified in four studies, representing a combined 626 participants), such that individuals who report intense pain in the limb before undergoing an amputation are also more likely to report intense postamputation PLP. Mechanistically, the positive relationship between preamputation limb pain and PLP (as well as RLP) suggests there may be shared pain elements. There is a longstanding body of evidence demonstrating the positive relationship between chronic preoperative pain predicting postoperative acute^41 50^ and chronic pain.^25 51 52^ Several mechanisms have been proposed for how pain at one timepoint triggers molecular alterations throughout the peripheral nerve, spinal cord and central nervous system that increase vulnerability to develop pain in the future.^39^ Other mechanisms for high association between preamputation pain and PLP could be genetic (eg, pain genes)^53^ or psychobiological vulnerability factors associated with increased susceptibility to chronic pain. While these may all be important contributors to the presence and severity of PLP, it is important to consider that many individuals who do not report pain before amputation still develop PLP.

Preamputation limb pain is a critically important factor for clinicians to consider when evaluating whether an elective amputation is a viable treatment for chronic neuropathic limb pain.^54–56^ The primary study suggesting elective amputation to be an effective treatment for chronic limb pain^55^ suffers from severe methodological problems, including retrospective reporting of preoperative pain on average 3 years after the amputation and lack of adequate controls (a pervasive methodological issue that we will discuss later). In contrast, a prospective, longitudinal survey of 31 patients who underwent elective amputation to treat chronic regional pain syndrome found that preamputation pain was the best predictor of postamputation pain.^57^ This evidence combined with the cross-sectional PLP studies identified in the meta-analysis suggests that preamputation limb pain will likely persist after amputation and re-present itself as PLP.

#### Non-painful phantom sensations

In the meta-analysis, non-painful phantom sensations comprised the third most common factor positively associated with PLP (identified in three study samples, representing a combined 1084 participants). Approximately 80% of amputees report experiencing some form of these non-painful phantom sensations,^1 14 23 28 35 58–61^ which range from kinetic (perception of passive or active phantom movement), kinaesthetic (awareness of phantom’s size, shape, position) to exteroceptive (tactile, pressure, temperature, etc). Most individuals describe phantom sensations as the feeling that the missing limb is still present and include feelings of tingling, itching, paresthesias or even a numb sensation, described as if the limb is asleep (figure 1B).^23 59^ In our experience, the boundary between non-painful and painful sensations in amputees can be blurry, including the adjectives used to describe them. For example, two amputees could report similar ‘itching’ phantom sensations. Yet, one of these amputees might characterise their ‘itching’ sensations as non-painful and the other as bothersome or even painful. Indeed, both painful and non-painful phantom sensations have been suggested to exist on a continuum of intensity, with the former more intense than the latter.^62^ This potential for vagueness needs to be methodologically considered and addressed when characterising PLP in an amputee population. For example, it is important to use specific adjectives that have been proven to reliably describe the experience of sensations versus pain or salience ratings (eg, MPQ). However, issues of translation aside, the same descriptives and scales could still be interpreted differently by individuals.

A form of non-painful phantom sensations that have been linked with PLP relates to kinetic sensations, which includes the ability to voluntarily move the phantom limb and, in some cases, non-voluntary phantom movements (for a review see^63^). There is some evidence supporting a relationship between the ability to volitionally move the phantom limb and lower PLP intensity.^64 65^ Other research highlights a positive relationship between persistent cortical representation of the phantom hand (elicited by instructing amputees to move their phantom hand) and severity of chronic PLP.^66 67^ This is consistent with the previously discussed association with peripheral contributions to the persistence of PLP. While there is currently no consensus on how cortical sensorimotor hand representation might mechanistically modulate pain processing, it has long been suggested that PLP is related to amputation-mediated maladaptive brain plasticity of the neural organisation supporting the body. In direct contrast to the peripheral account described above, this theory proposes that loss of peripheral input after amputation causes PLP due to neural changes to the somatosensory body representation, where the brain territory that once supported the amputated body part is subsequently deprived of input and might even become activated by neighbouring body parts.^68–70^ While today it is agreed that there is no simple relationship between somatosensory map reorganisation and PLP,^71^ the maladaptive plasticity theory dominated contemporary research on PLP for several decades. This theory provided clear predictions on how to treat phantom pain: if pain is caused by maladaptive reorganisation, then we need to reverse it to alleviate phantom pain. In that vein, several treatment approaches attempt to restore the representation of the missing hand by increasing the phantom hand’s motor control (either directly via phantom training or indirectly by illusory visual feedback of the missing hand) with the hope it will decrease PLP (eg, graded motor imagery (GMI) and mirror therapy (MT)).^64 72–75^ As causal evidence has yet to be published showing the ability to move the phantom leads to lower PLP intensity, this complex association between PLP and phantom movement sensations further fuels the need to develop a more sophisticated understanding of the potential mechanistic association between PLP and other related phantom sensations.

#### Time since amputation

Finally, one factor, not identified by the meta-analysis, that we think is worth discussing because it is so frequently reported and commonly thought to be associated with PLP is time since amputation. Indeed, many individuals want to know whether their PLP and/or RLP will resolve with time. While certain cross-sectional and retrospective studies have suggested there is a gradual decrease in PLP over time,^61 76 77^ this evidence relies on retrospective recall of pain intensity,^14^ which is known to be unreliable.^78^ For example, retrospective pain memory is known to contain positivity bias for more recent experiences.^79 80^ In addition, there’s concern for a selection bias, where amputees who are no longer bothered by PLP will be less motivated to take part in PLP surveys. However, from prospective longitudinal studies, at least up to 3.5 years post amputation, there is evidence to suggest that PLP prevalence or intensity scores remain constant (figure 3).^25 40 41 81–84^ To get a sense of the attrition bias in the studies, the literature would benefit from future studies that compare initial pain scores between participants who provided full follow-up data and those who dropped out or were lost to follow-up. However, at present, the evidence from prospective longitudinal studies shows that PLP prevalence or intensity remains relatively constant over time. The same appears to be true for the long-term natural time course of RLP (beginning 6 months after amputation once the residual limb has healed).

While a recent cross-sectional survey of 727 individuals found that those within 1 year of amputation have greater odds of reporting PLP than those whose time since amputation was more than 10 years (OR, 1.86; 95% CI, 1.19 to 2.89; p=0.01^30^), other large-sample cross-sectional studies (sample with 536 amputees^14^ and sample with 914 amputees^85^) did not find an association between PLP and time since amputation. Combined with the multiple issues relating to use of multiple PLP measures and selection bias, as well as other factors associated to time since amputation, such as age at amputation, there is little evidence to suggest that PLP resolves with time. Instead, the available evidence suggests that PLP and RLP remain relatively constant, at least up to 2 years after amputation. Understanding that PLP does not change with time is crucial for managing patient expectations.

To summarise, PLP is clearly a highly prevalent and heterogenous phenomenon in amputees. Developing a better understanding (and quantification) of PLP covariates is necessary for better handling of this heterogeneity in future research and for providing valuable clues as to the underlying mechanism(s) of PLP. From a therapeutic perspective, we need to understand whether the observed associations between PLP and the amputation-related factors mentioned above actually hint at potential causality (proven only through direct experimental manipulation). This understanding is essential when evaluating PLP treatments used today that are inspired by these observed associations. In the next section, we will build on this background on PLP heterogeneity and known covariates to evaluate the rationale of PLP treatments and, critically, the research practices behind them.

## PLP treatments

**Figure 4:**
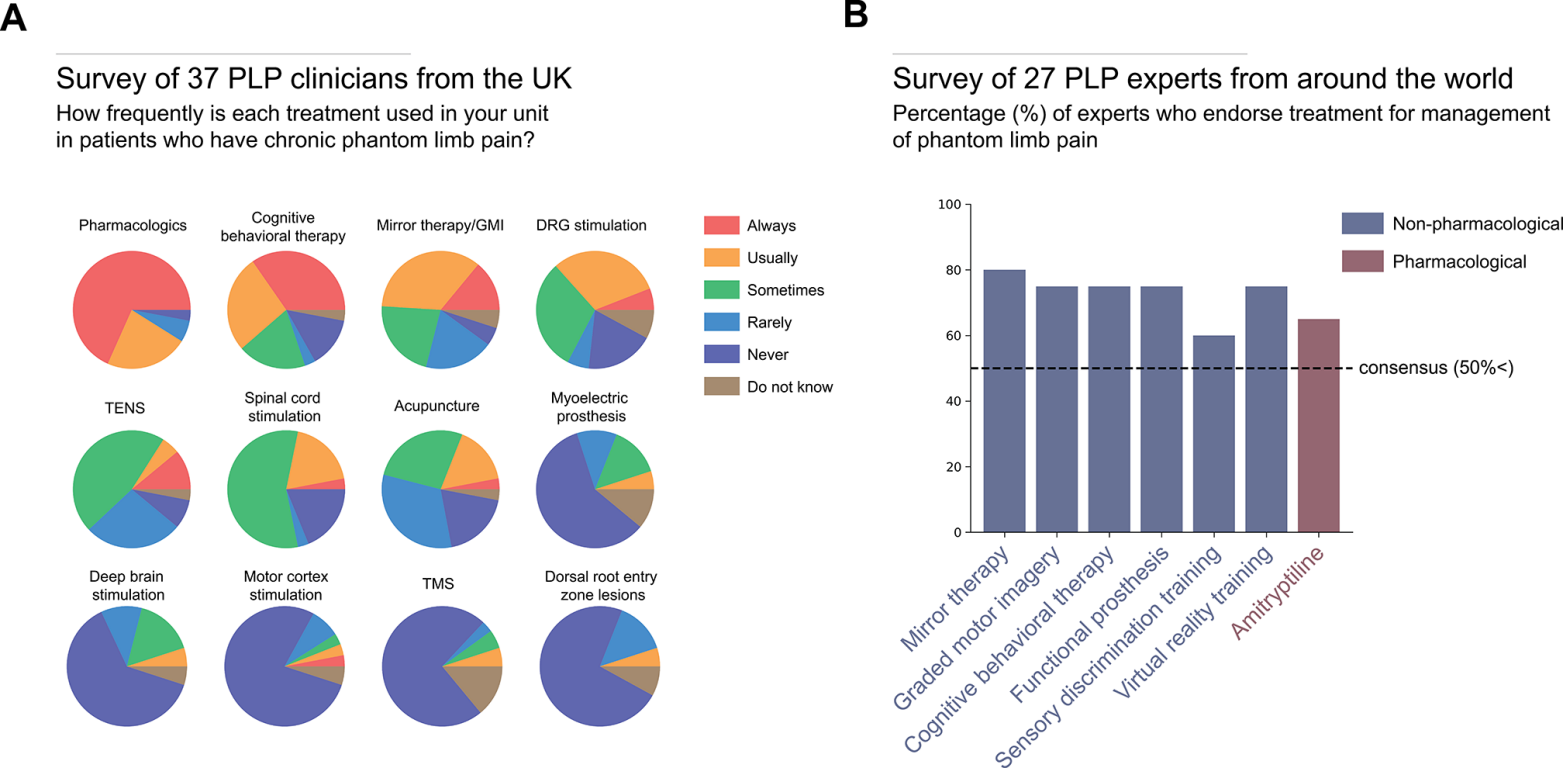
Therapies and treatments for phantom limb pain (PLP). (A) A recent survey of 37 PLP clinicians, 23 pain physicians, 11 neurosurgeons, 2 anaesthetists and 1 rehabilitation physician, across 30 UK hospitals, revealed that most clinicians are primarily using pharmacological treatments for chronic PLP patients, as well as a combination of non-pharmacological and brain stimulation therapies. Data adapted from Table 27 in Corbett *et al* 4. (B) A global Delphi review of PLP treatments used by PLP experts, three anaesthetists, three physiatrists, two psychologists, two neurologists, eight physiotherapists, one nurse and one occupational therapist, revealed that most consensus treatments were non-pharmacological. Graded motor imagery (GMI); transcutaneous electrical nerve stimulation (TENS); transcranial magnetic stimulation (TMS). Data adapted from Table 2 in Limakatso and Parker^3^ with permission.

In 1983, nearly 40 years ago, Sherman and Sherman catalogued more than 60 potential viable PLP treatments. Since then, we still have not converged on a consensus.^86^ For example, a recent survey of 37 pain physicians and neurosurgeons based across 30 UK pain clinics found that 12 different pharmacological and non-pharmacological therapies were being used to treat PLP, with pharmacological treatments being the most popular class of PLP intervention (figure 4A).^4^ While the report did not specify which pharmacologics were used, in our experience, the most common pharmacological agents used to treat PLP are gabapentin,^87–89^ pregabalin and amitriptyline. Despite contradicting evidence from randomised control trials (RCTs) for the efficacy of these treatments for PLP, most clinicians continue to use them (for reviews on the RCTs testing the efficacy of pharmacological treatments on PLP, see^7 8 90^).

In another survey across 16 countries, Limakatso and Parker^3^ asked 27 clinicians and researchers which PLP treatments are viewed to be effective.^3^ Out of a total of 37 PLP treatments, there was a majority consensus (>50%) on seven to be effective at reducing PLP, 6 of which were non-pharmacological (figure 4B). At least four of these interventions are behavioural, specifically designed to target central contributors to PLP by attempting to restore the original representation of the missing limb by reversing the maladaptive plasticity that has presumably been triggered by the sensory and motor limb loss. These treatments aim to achieve this reversal through: restoring visual feedback of the missing limb (MT),^91^ mental imagery exercises to strengthen the representation of the phantom limb (graded motor imagery),^92^ increasing the representation of the residual limb via perceptual learning (sensory discrimination training)^93^ and combining these approaches to improve the executing of phantom movements via virtual reality^94^ or augmented reality.^74^ While these techniques might reduce reliance on medication, it is important to critically consider not only whether they have clinical efficacy but also whether, and in what way(s), they influence the mechanisms underlying PLP.^95^

**Figure 5:**
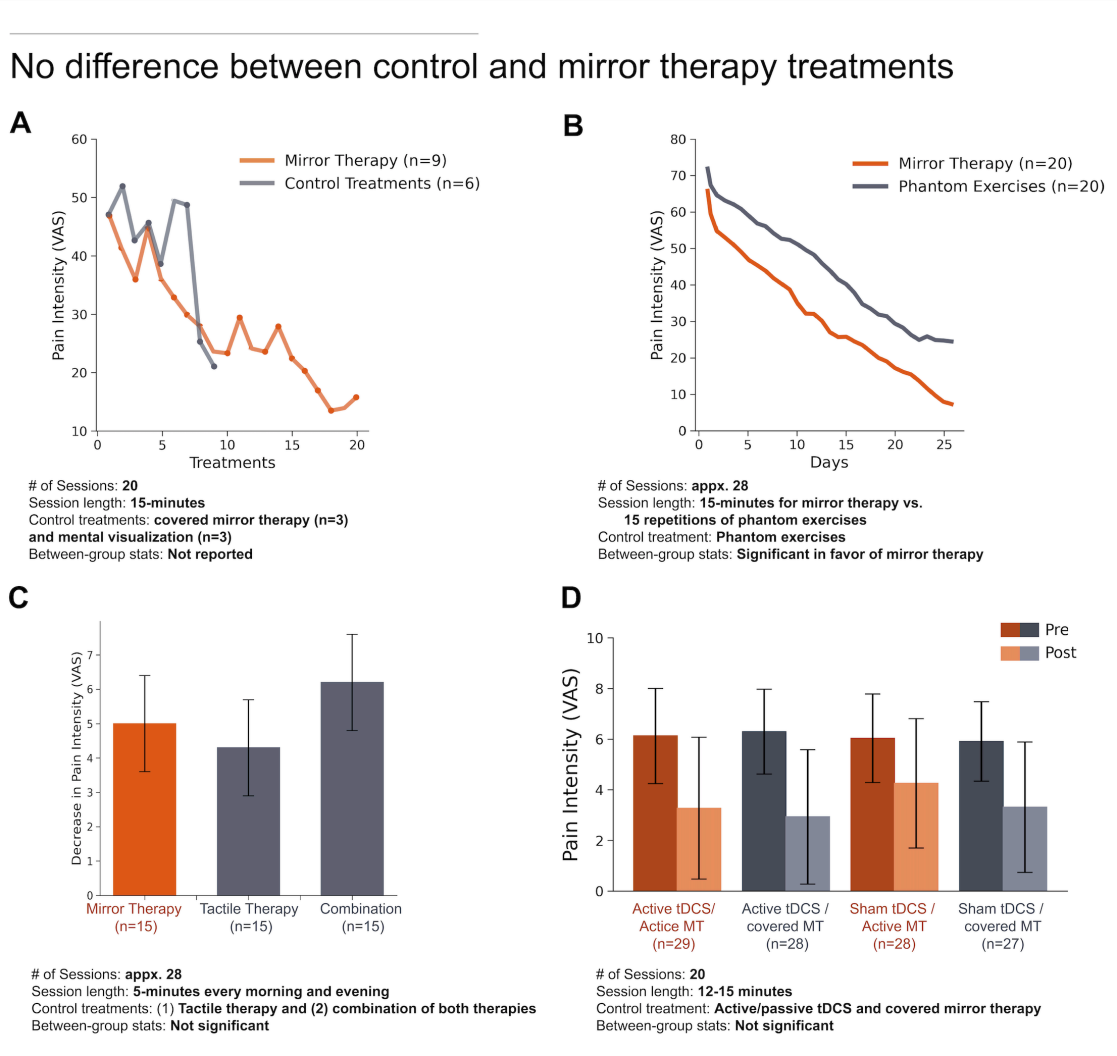
Recent studies of mirror therapy (MT) for PLP show a lack of efficacy when compared with various control therapies. (A) Randomised control trial (RCT) comparing MT to control treatments (combined data from three amputees assigned to a covered MT treatment and three amputees assigned to perform mental visualisation exercises). Data adapted from figure 2 in Finn et al^100^ with permission. (B) RCT comparing MT to phantom exercises. Data adapted from figure 2 in Anaforoğlu Külünkoğlu et al^101^ with permission. (C) RCT comparing MT to tactile therapy and a combination therapy treatment which included a serial combination of mirror and tactile therapy. Y-axis reflects decease in magnitude of VAS pain intensity before and after 4 weeks of therapy. Data adapted from table 2 in Ol et al^102^ with permission. (D) A randomised, blinded, sham-controlled, 2×2 factorial clinical trial comparing transcranial direct current stimulation (tDCS) and MT. Participants were placed into 1 of the 4 treatment groups: (1) active tDCS and active MT, (2) active tDCS and covered MT, (3) sham tDCS and active MT and (4) sham tDCS and covered MT. Data adapted from figure 3 in Gundez et al^103^ with permission. PLP, phantom limb pain; VAS, Visual Analogue Scale. (A, B) Scale is 0–100 mm. (C, D) Scale is 0–10 mm.

For example, mirror (box) therapy is a highly popular cognitive treatment according to both expert consensus surveys, more so than any other non-pharmacological treatment of PLP. A recent systematic review by Aternali and Katz^5^ of recent RCTs concluded that there is no difference in PLP outcomes between MT and various control therapies (figure 5).^5^ Indeed, administering treatment of any kind may be sufficient to show a short-term reduction in PLP, even in patients who have shown resistance to past treatments. This could be a consequence of taking part in the study (eg, engagement, attentional distraction)^96^ or interactions with the experimenter (eg, compliance, suggestion).^97^ Unlike studies evaluating pharmacological treatments which are more conducive to placebo-controlled double blinding, behavioural interventions are particularly susceptible to these placebo effects (eg, similar decreases in PLP from control treatments), because they are often designed to make the therapy more engaging (eg, virtual reality) and because these experiments have not been, and often cannot be, designed in a double-blinded manner (only the participant is kept blinded), which is not sufficient to protect a study from expectation bias.^97^ In the absence of a randomised, double-blind, placebo-controlled design, observed changes in PLP reports may be due to one or more uncontrolled factors and not necessarily the putative mechanism being tested.

So, what can be done to improve the level of evidence for the efficacy of behavioural treatments and their underlying mechanism(s)? A crucial first step in study design is to choose an appropriate control intervention that isolates the active component(s) thought to be responsible for the treatment effect and administer it to the treatment group only while ensuring that all other aspects associated with treatment are provided to both the treatment and control groups. For example, with mirror box therapy, the putative active components are the combined visual input of seeing the (amputated) extremity restored along with the synchronous movements of the phantom and contralateral intact extremities, therefore the control condition(s) should include all other aspects of the task, but mismatch either the visual feedback (eg, covered mirror) or ask participants to perform asynchronous phantom and hand movements while looking in the mirror. Second, the experimenter needs to consider other aspects of the treatment that might be indirectly modulating the pain experience. For example, the visual mismatch might reduce the level of task engagement, risking a ‘contaminated’ control (eg, less engagement for one control therapy compared with the active therapy). Third, it is important that both experimenter and patient should have no treatment preference, between the main treatment and the control treatment, a concept known as clinical equipoise.^98^ Here, the concern is that prior knowledge about mirror treatment will further influence the study. Practically speaking, these concerns are not easy to address, but they are addressable. For example, experimenters should work to creatively design control therapies that can both control for the active component as well as maintain a similar level of participant engagement. Further, experimenters should carefully design how active and control interventions are administered (eg, double blinding when possible). With regards to randomisation of participants across treatments, especially when considering the small samples used in nearly all these studies, it is important to make sure that baseline differences in PLP experience are not impacting the treatment, including not just pain intensity but also frequency and other phantom phenomenology which might be relevant for treatment outcome (eg, RLP; the level of mobility of the phantom; pain disposition). One approach to accomplish this is to use stratified random sampling based on known associated factors. This will ensure an equal number of participants with each characteristic (strata) in all groups. Finally, when analysing the data, it is important to account for PLP covariates, even if these do not differ statistically across groups at baseline.

In summary, on the treatment front, there is still no sufficient strong evidence to support the efficacy of any PLP treatment. Interestingly, in the Limakatso and Parker^3^ survey, only ~25% of the clinical experts endorsed the statement that there is strong scientific evidence supporting the effectiveness for any one of these consensus treatments.^3^ As such, there seems to be a discrepancy between the enthusiasm found in research and the scepticism shown in the clinic. One potential reason for this gap is that the individual studies assessing treatment efficacy are often plagued with methodological problems, which will consequently limit clinical impact. Given the abovementioned concerns for the quality of the primary evidence for many of these treatments, a re-evaluation is necessary of the research practices and standards we hold ourselves to when assessing the efficacy of a treatment.

### Recommendations for evaluating and planning research on PLP

**Figure 6:**
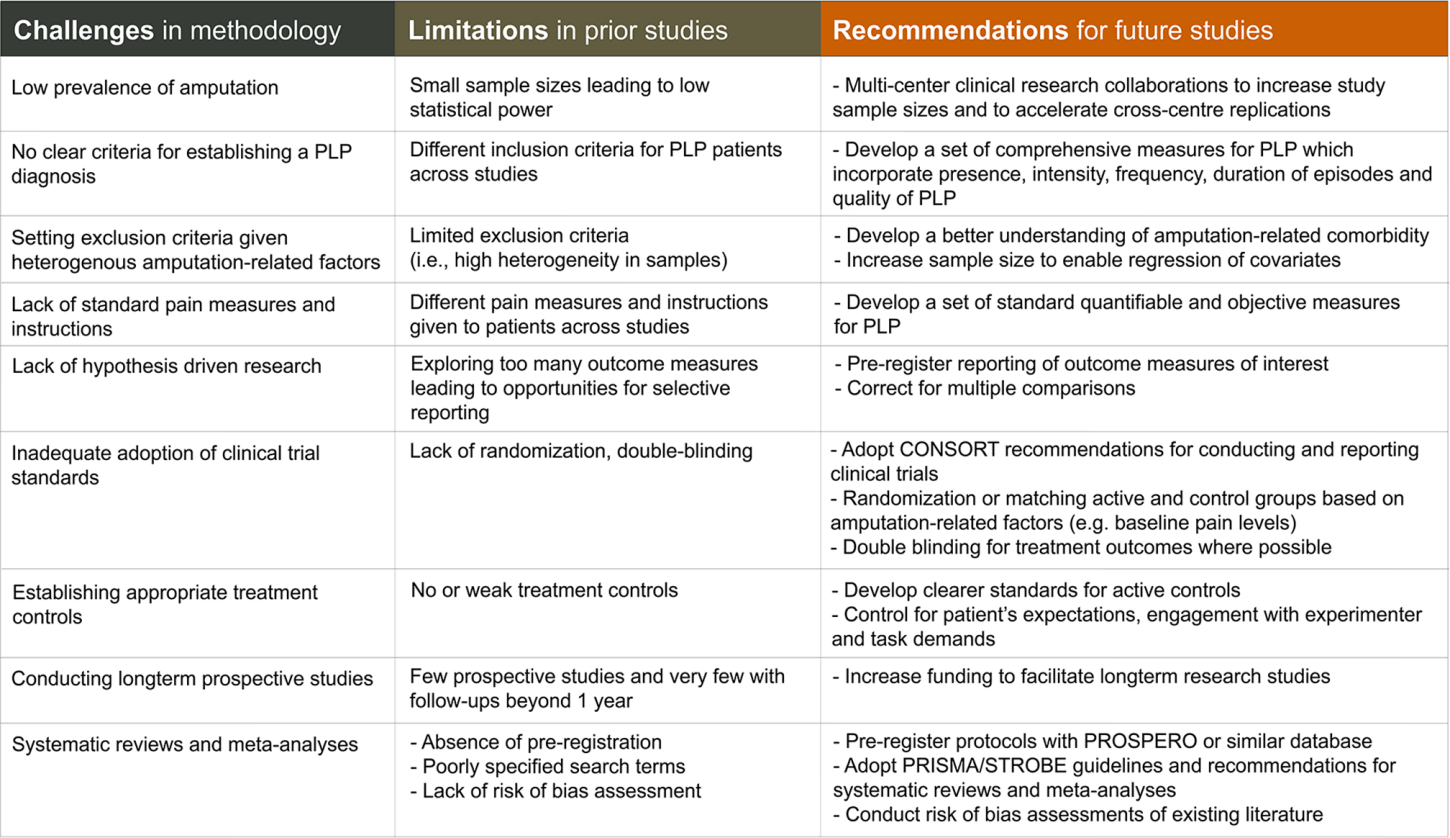
Challenges, limitations and recommendations for phantom limb pain (PLP) research. The methodological challenges with studying PLP have hindered the accumulation of reliable information on PLP. For each of these, we provide specific examples for how they might impact the existing literature and recommendations that could be adopted to help overcome each challenge. CONSORT, Consolidated Standards of Reporting Trials; PRISMA, Preferred Reporting Items for Systematic Reviews and Meta-Analyses; STROBE, Strengthening the Reporting of Observational Studies in Epidemiology.

Since Sherman and Sherman’s catalogue of PLP treatments, nearly 40 years ago, research endeavours to develop and evaluate PLP treatments have been largely trial and error.^86^ One potential reason for this is that PLP is very difficult to quantify, considering its heterogenous phenomenology and covariates. Other reasons are methodological including small sample sizes, inappropriate or absent control conditions and differences across studies in how treatments are administered and the outcome measures used to test them. In addition, researchers are typically not being selective when they recruit individuals to take part in PLP studies, either because they do not know what to select for (eg, level of amputation, number of amputations, nature of phantom limb: clenched, paresthesia/dysesthesia, etc) or because people with amputations are simply too difficult to recruit so researchers implement minimal exclusion criteria and enrol whomever they can. While these challenges (summarised in figure 6) have slowed the accumulation of reliable knowledge on PLP, we are hopeful that corrective actions to overcome these conceptual and methodological hurdles will lead to the development of more meticulous assessment techniques and effective treatments.

To summarise the more important considerations, when evaluating the reliability of already published PLP research, we recommend conducting a risk of bias assessment using any one of a number of available tools^99^ to evaluate the degree of bias in relevant study domains: the sample size, heterogeneity of the sample and the potency of the treatment controls used. Similarly, when interpreting findings from a heterogenous amputee sample, it is important to consider the potential effects of PLP covariates (eg, RLP, preamputation pain, non-painful phantom sensations) on the study’s findings. Reviewers should insist on preregistration of key outcome measures, double blinding of treatment versus controls (when possible) and more careful interpretation of exploratory analysis, to improve the solidity of the reported findings. When planning future research on PLP, we strongly recommend that researchers recruit larger and more homogeneous samples (based on amputation-related factors). Further, researchers should prioritise collecting long-term prospective data and conducting carefully considered RCTs with potent treatment controls. Considering the practical difficulties with implementing these recommendations, we feel it is crucial that researchers and clinicians build multicentre clinical research collaborations that will allow for either large-scale samples or replications across independent patient samples. Additionally, we feel strongly that we need to develop standardised quantifiable measures for PLP. This will not only reduce the practice of ‘p-hacking’ when analysing results but will also provide a much-needed opportunity to compare findings across studies. Beyond the presence and intensity of PLP, such measure should take into consideration, frequency (daily, weekly, etc), duration of episodes (seconds, minutes, hours) and impact on quality of life.

While this list is not exhaustive, we hope that these considerations are helpful for researchers and clinicians when evaluating already published PLP research and when planning future research. Our aspiration is that more reliable evidence will enable clinicians to make more informed decisions about available and effective treatments and will guide the development of the next generation of PLP treatments away from unnecessary pitfalls and help accelerate the development of more effective treatments.
